# Reversible impairment of non-invasively assessed mitochondrial oxygen metabolism in the long-term course of patients with sepsis: a prospective monocentric cohort study

**DOI:** 10.1186/s40635-025-00808-x

**Published:** 2025-11-14

**Authors:** Anne Standke, Charles Neu, Philipp Baumbach, Alina K. Plooij, Kornel Skitek, Juliane Götze, Sina M. Coldewey

**Affiliations:** 1 Department of Anaesthesiology and Intensive Care Medicine, Septomics Research Centre, Translational Septomics, Jena University Hospital, Friedrich-Schiller-University Jena, Jena, Germany; 2 Institute of Anaesthesiology and Perioperative Medicine, University Hospital Zürich, University of Zürich, Zürich, Switzerland

**Keywords:** Cellular oxygen metabolism monitor, Mitochondrial oxygen tension, Mitochondrial oxygen consumption, Mitochondrial oxygen delivery, Protoporphyrin IX-triplet state lifetime technique (PpIX-TSLT)

## Abstract

**Background:**

Sepsis is characterized by organ dysfunction due to infection, with increasing evidence of mitochondrial dysfunction assessed preclinically and invasively. Protoporphyrin IX-triplet state lifetime technique (PpIX-TSLT) permits non-invasive determination of cellular oxygen metabolism and may provide deeper pathophysiological insights.

**Methods:**

This analysis is part of a prospective monocentric cohort study. ICU patients with sepsis and septic shock and healthy controls were enrolled between May 2018 and June 2022. Mitochondrial oxygen tension (mitoPO_2_), consumption (mitoVO_2_) and delivery (mitoDO_2_) were assessed in the skin of healthy controls and patients with sepsis in the acute phase (3 ± 1 days after onset) and long-term course of disease (6 ± 2 months after onset) using PpIX-TSLT (CE-certified *Cellular Oxygen METabolism* system). Primary endpoints were differences in mitoPO_2_, mitoVO_2_, and mitoDO_2_ between patients in the acute phase of sepsis and controls. We tested group differences with t-tests and report Cohen’s d (d) as effect size.

**Results:**

In the acute phase, mitochondrial oxygen tension (mitoPO_2_) was significantly reduced (*n* = 133, mean ± standard deviation: 58.4 ± 19.2 mmHg) compared to controls (*n* = 79, 67.3 ± 17.7 mmHg, *p* = 0.002, *d* = − 0.48). We found no significant differences in oxygen tension in the long-term course (*n* = 43) or in oxygen consumption and delivery between acute and long-term course of sepsis and controls. In the acute phase, lower mitochondrial oxygen delivery was associated with higher Sequential Organ Failure Assessment score (Spearman’s *ρ* = − 0.23, *p* = 0.009) and higher lactate concentrations (*ρ* = − 0.21, *p* = 0.021) and, thus, correlated with disease severity.

**Conclusions:**

Our results suggest that cellular oxygen metabolism in sepsis is characterized by a reversible restriction of oxygen tension without an impairment of mitochondrial oxygen consumption. Additionally, oxygen delivery is dependent on disease severity. These findings should be re-validated in a larger cohort.

**Trial registration:**

NCT03620409 (Ethics vote: 5276-09/17; German Register of Clinical Studies: DRKS00013347), Principal investigator: Sina M. Coldewey, Date of Registration: 11-30-2017 NCT03620409

**Supplementary Information:**

The online version contains supplementary material available at 10.1186/s40635-025-00808-x.

## Background

Sepsis is characterized by life-threatening organ dysfunction due to a dysregulated host response to infection [1]. Targeted molecular therapies and parameters for early prognostication are still lacking, although pathophysiological understanding has improved preclinically [2]. Changes in blood lactate concentration [3] and microcirculatory blood flow [4–6] have been described as prognostic markers of sepsis. However, these markers cannot distinguish between tissue hypoxia and disturbed oxygen utilization (dysoxia). There is increasing evidence of mitochondrial dysfunction in sepsis using invasive or ex vivo techniques [7–10].

Measuring mitochondrial function non-invasively and directly at the bedside remains challenging, but has the advantage of allowing repeated measurements and eliminating potential confounding factors of invasiveness [11]. Based on Protoporphyrin IX-Triplet State Lifetime Technique (PpIX-TSLT), Mik and colleagues developed a measurement system to assess cellular oxygen metabolism in the epidermis, non-invasively and in vivo [12–15].

The measurement of mitochondrial oxygen tension (mitoPO_2_) in the skin using this technique has been validated in both preclinical studies and in healthy subjects [16, 17]. Growing evidence suggests that the skin, like the intestines [18, 19], can serve as a sentinel organ for disturbances in systemic oxygen metabolism in certain conditions [12, 20–23]. A haemodilution experiment in pigs, conducted by Römers and colleagues, did show a decrease in mitoPO_2_ prior to alterations in venous saturation and blood lactate concentration [20]. Patients who experienced prolonged periods of low mitoPO_2_ intraoperatively were more likely to develop postoperative acute kidney injury [21, 22]; whereas, mean arterial pressure and peripheral oxygen saturation showed no association with the development of acute kidney injury [22]. Werfers Bettink and colleagues administered lipopolysaccharide (LPS) to healthy volunteers to induce systemic inflammation. They observed a significant decrease in mitoPO_2_, reaching its nadir after 1.45 h. In contrast, the highest heart rate and lowest mean arterial pressure were recorded 4 h after LPS administration [23]. These studies underscore the potential of non-invasively measured mitoPO_2_ as an early and sensitive indicator that may offer valuable insights into the perfusion status of internal organs. The feasibility of PpIX-TSLT using the Cellular Oxygen METabolism (COMET) system in the acute phase of sepsis has been demonstrated by a pilot study [24]. To the best of our knowledge, the cellular oxygen metabolism has not yet been investigated in the long-term course of sepsis.

The primary aim of this study was to non-invasively characterize cellular oxygen metabolism in patients with sepsis and to investigate its alterations in the acute course of sepsis compared to controls. In addition, its prognostic relevance for mortality, changes over the course of the disease, and associations with demographic and clinical variables were investigated.

## Methods

The analysis is part of the prospective, monocentric study ‘Identification of cardiovascular and molecular prognostic factors for the medium-term and long-term outcomes of sepsis’ (acronym: ICROS, [25]). Detailed information on the study design, sample size calculation and methodology are provided in the published study protocol [25] and Supplementary Methods (Supplementary Material). The study was approved by the Ethics Committee of the Friedrich Schiller University Jena on October 10, 2017 (5276-09/17) and is registered (ClinicalTrials.gov: NCT03620409 and German Register of Clinical Studies: DRKS00013347).

### Patient sample

Patients with sepsis or septic shock according to the sepsis-3 definition in the intensive care units of the Jena University Hospital were enrolled in the ICROS-study between May 2018 and March 2021 [25]. The healthy controls were recruited in a target age range and sex proportion similar to that of patients with sepsis. Written informed consent was obtained from either the patients or their legal surrogates and all controls. The PpIX-TSLT measurements from 37 patients in the acute phase of sepsis are published as a feasibility study [24].

### Study design

Briefly, PpIX-TSLT measurements were performed at 3 ± 1 days (T1, acute phase) and during a follow-up at 6 ± 2 months (T4, long-term course) after sepsis onset. Baseline data (e.g. demographics) were collected at study enrolment. Clinical variables and laboratory samples were collected at all time-points. Patients were contacted by telephone to arrange follow-up visits, or telephone interviews were conducted if patients were unable to attend the hospital (e.g. due to poor health or COVID-19 restrictions). In controls, laboratory sampling and PpIX-TSLT measurement were performed once at study enrolment.

### Assessment of cellular oxygen metabolism

All measurements were performed with the CE-certified Cellular Oxygen METabolism (COMET) system (Photonics Healthcare, Utrecht, Netherlands, [12]). Based on the PpIX-TSLT, it enables the non-invasive assessment of the mitochondrial oxygen tension (mitoPO_2_, mmHg) in the epidermis as described previously [12–15]. A transdermal laser pulse is used to excite PpIX, a precursor of haem in mitochondria, resulting in delayed fluorescence [13]. MitoPO_2_ is determined from fluorescence lifetime, which is inversely proportional to oxygen tension [14].

To enrich PpIX, a 4-cm^2^ patch containing 5-aminolevulinic acid (ALA, Alacare^®^, 8 mg, photonamic, Wedel, Germany) was applied to shaved, cleaned and dried skin at least four hours before the scheduled measurement to ensure sufficient signal quality. According to the standardized measurement protocol, in the first 30 s, mitoPO_2_ was measured by applying the sensor to the skin. Subsequently, mitochondrial oxygen consumption (mitoVO_2_, mmHg/s) was measured by applying pressure to the sensor, which interrupts the microcirculation until the oxygen is depleted (approximately 45 s). After the release of pressure, blood inflow reoxygenates the tissue (capillary refill, approximately 30 s) and mitochondrial oxygen delivery (mitoDO_2_, mmHg/s) was determined. The measurement was taken in the clavipectoral triangle, a location that is less susceptible to temperature changes, movement and peripheral vasoconstriction. The proximity of bony structures allows for effective compression of the microcirculation, thereby ensuring a reliable measurement of mitoVO_2_ and mitoDO_2_, even in patients with obesity and severe overhydration. This procedure was repeated three times. Repeat measurement values were averaged before subsequent statistical analysis.

The data management, data preparation, and estimation of the PpIX-TSLT variables were performed using a self-developed script (Halley) in MATLAB (MATLAB, and Statistics Toolbox Release 2017a, The MathWorks, Inc., Natick, Massachusetts, United States). For details please see [15].

### Study endpoints

The primary endpoints were differences in the PpIX-TSLT variables (mitoPO_2_, mitoVO_2_, and mitoDO_2_) between the acute phase of sepsis (T1) and controls.

Secondary endpoints comprised:

- Differences in PpIX-TSLT variables between long-term course of sepsis (T4) and controls as well as longitudinal comparisons (T1 *vs* T4).
- Prognostic value of PpIX-TSLT variables in the acute phase (T1) for mortality (28 day and 180 day mortality).
- Associations of PpIX-TSLT (T1) with demographic (age, body mass index), clinical (heart rate, blood pressure, oxygen saturation), and laboratory variables (blood lactate concentration, bilirubin, haemoglobin), as well as severity (Sequential Organ Failure Assessment (SOFA) score, requirement of vasopressor therapy) and course of disease (length of stay in ICU and hospital, duration of vasopressor therapy).
- Associations of PpIX-TSLT variables (T1) with variables of the measurement environment (temperature of body, skin, room, and measurement sensor; duration of ALA-application).

### Statistical analysis

This study is exploratory in nature; thus, the results should primarily be regarded as hypothesis-generating. Depending on the distribution of the variables, descriptive statistics for continuous variables include means, standard deviations (SD), medians, and interquartile ranges (IQR). For dichotomous and categorical variables, we report absolute (n) and relative frequencies (%). Missing data were few and not imputed. The number of data points included in the specific analysis is indicated.

We applied independent samples t-tests to analyse group differences of the PpIX-TSLT variables (mitoPO_2_, mitoVO_2_, and mitoDO_2_) between patients at T1 and at T4 and controls (primary endpoints). P-values were adjusted with the Bonferroni–Holm correction.

Analyses of secondary outcomes were exploratory; p-values were not adjusted for multiple testing. Longitudinal changes in PpIX-TSLT variables (T1 *vs* T4) were analysed with paired t-tests. The prognostic value of mitoPO_2_, mitoVO_2_, and mitoDO_2_ at T1 for 28-day and 180-day mortality was analysed using independent samples t-tests (survivors *vs* non-survivors). To adjust for age, sex, comorbidities (Charlson comorbidity index), and disease severity (SOFA score), we additionally applied logistic regression. Mortality was modelled as dependent variable; mitoPO_2_, mitoVO_2_, and mitoDO_2_ were modelled separately as independent variables. We report adjusted odds ratios (OR) and 95% confidence intervals (95%-CI).

The associations of the PpIX-TSLT variables in the acute phase (T1) with continuous demographic, clinical and laboratory variables as well as continuous variables of disease severity, course of disease, and the measurement environment were analysed with Spearman’s rank correlation coefficients. Associations with dichotomous variables (grouping variables, e.g. sex) were analysed with independent samples t-tests.

Finally, independent samples t-tests were used to assess differences in PpIX-TSLT variables at T1 between patients with vasopressor therapy and those without.

The statistical analyses were performed using SPSS (Version 27.0. IBM, Armonk, NY: IBM Corp, USA), and R (Version 3.5.1. Vienna, Austria). We report two-sided p-values and consider p < 0.05 as significant. Where applicable, we report Cohen’s d as effect size (|d|= 0.2 small effect; |d|= 0.5 medium effect; |d|= 0.8 large effect, [26]).

## Results

### Sample characteristics

**Fig. 1:**
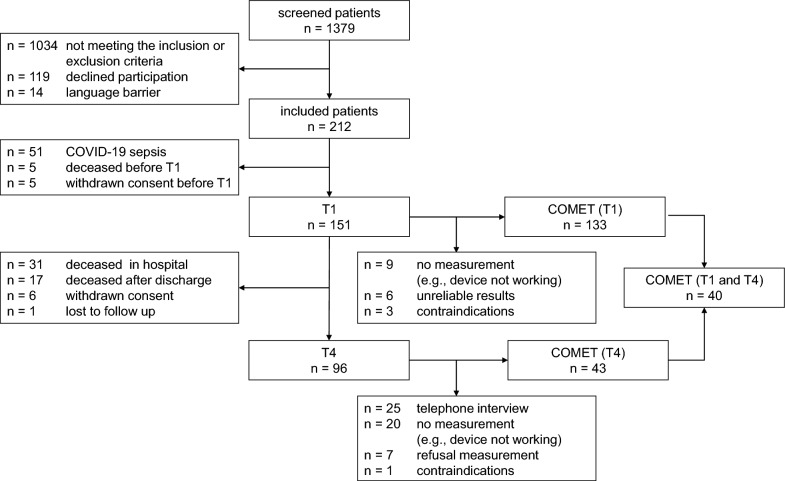
Overview of the enrolled patients and analysis cohorts. In addition, 81 controls were enrolled and 79 were included in the statistical analysis

Figure 1 summarizes the enrolment, exclusion, and resulting analysis cohorts. The demographic and clinical characteristics of patients with a PpIX-TSLT measurement at T1 or T4 (*n* = 136) are summarized in Table 1. Patients and controls (*n* = 79) did not differ in age (median [IQR] 66 [56, 75] *vs* 65 [52, 72] years; *p* = 0.413) or sex (female 34.6% (47/136) *vs* 36.7% (29/79); *Χ*^2^ (1, *N* = 215) = 0.03, *p* = 0.865). The body mass index was significantly higher in patients (27.8 [23.7, 32.4] kg/m^2^) than in controls (25.5 [23, 27.2] kg/m^2^; *p* < 0.001). Primary site of infection was pneumonia (50%).

**Table 1:** Demographic, laboratory and clinical characteristics of the patients with at least one PpIX-TSLT measurement

Variable	*n* = 136 patients
Age, years	66 [56, 75]
Female sex	47 (34.6%)
Body mass index, kg/m^2^	27.8 [23.7, 32.4]
Charlson comorbidity index, points	2 [1, 4]
Site of infection	
Pneumonia/respiratory	68 (50%)
Intra-abdominal/gastrointestinal	48 (35.3%)
Urogenital	19 (14%)
Bone/soft tissue	14 (10.3%)
Others^*a*^	23 (16.9%)
Multiple foci	33 (24.3%)
Type of referral	
Surgical emergency	51 (37.5%)
Non-surgical emergency	46 (33.8%)
Elective treatment	39 (28.7%)
SOFA at study enrolment, points	9 [7, 11]
Septic shock at sepsis onset, yes	66 (48.5%)
Mortality	
28 day mortality	21 (15.4%)
180 day mortality	37/134 (27.6%), *n* = 2 censored
LOS in ICU, days	9 [4, 22], *n* = 110 survivors
LOS in hospital, days	30 [20, 49], *n* = 110 survivors
Duration of vasopressor therapy, days	6 [3, 14], *n* = 106 survivors^†^
Laboratory variables	
CRP (max), mg/l	200.8 [126.7, 275.8], n = 134
Procalcitonin (max.), ng/ml	2.6 [1, 12.3], *n* = 135
Leukocytes (max.), Gpt/l	13 [8.8, 18.1], *n* = 135
Haemoglobin (min.), mmol/l	5.2 [4.8, 5.9], *n* = 135
Bilirubin (max.), μmol/l	13 [6, 26], *n* = 132
Lactate (max.), mmol/l	1.4 [1.1, 1.9], *n* = 129

^†^Survivors with vasopressor therapy (in total 132/136 (97.1%) with vasopressor therapy)

^*a*^Thoracic, surgical, intracerebral infections, and bacteraemia

Descriptive statistics include medians and interquartile ranges (IQR, continuous variables) and absolute (n) and relative frequencies (%, dichotomous and categorical variables). In case of missing data, the number of data points included in the specific analysis is indicated. SOFA, Sequential Organ Failure Assessment Score; LOS, length of stay; ICU, intensive care unit

At T1 and T4 95.7% (133/139) and 100% (43/43) of the PpIX-TSLT measurements were usable. In controls, 98.8% (79/80) of the PpIX-TSLT measurements were analysable. We did not observe any side-effects of the measurement or the ALA patch.

### Group differences of PpIX-TSLT variables in the acute and long-term course of sepsis

**Fig. 2:**
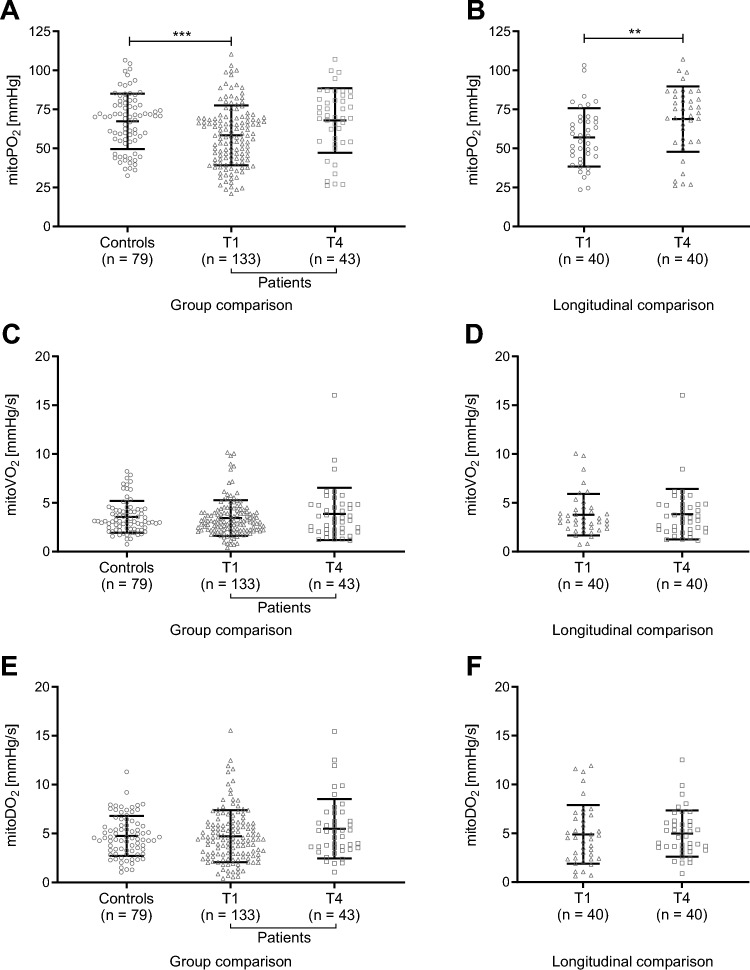
PpIX-TSLT variables of patients with sepsis and controls. Measurements were taken 3 ± 1 days (T1) and 6 ± 2 months after the diagnosis of sepsis (T4) and in healthy controls once at study enrolment. Mean and standard deviation of mitochondrial oxygen tension (mitoPO_2_, **A**, **B**), consumption (mitoVO_2_, **C**, **D**), and delivery (mitoDO_2_, **E**, **F**) are shown. Group differences between patients at T1 and at T4 and controls (**A**, **C**, **E**) were analysed with two-sided t-test for independent samples. Longitudinal changes in PpIX-TSLT variables (**B**, **D**, **F**) were analysed with two-sided t-test for paired samples. Significant differences are presented as follows: *p < 0.05, **p < 0.01, and ***p < 0.001

Compared to controls, mitoPO_2_ was significantly reduced in the acute phase of sepsis with small to medium effect size (mean ± SD; 58.4 ± 19.2 *vs* 67.3 ± 17.7 mmHg; *p* = 0.002; *d* = − 0.48; Fig. 2A; Supplementary Table 1). Longitudinal analysis revealed a significant increase of mitoPO_2_ between T1 and T4 (*n* = 40) with a small to medium effect size (57.1 ± 18.7 *vs* 68.8 ± 21.0 mmHg; *p* = 0.006; *d* = − 0.46; Fig. 2B; Supplementary Table 2). The individual trajectories of patients between T1 and T4 are visually illustrated in Supplementary Fig. 1. At T4, mitoPO_2_ did not differ between patients with sepsis (*n* = 43; 67.9 ± 20.7 mmHg; *p* = 0.994) and controls. A dropout analysis showed no differences in T1 PpIX-TSLT variables between patients with a measurement at T4 and those without (Supplementary Table 3). We found no significant differences between patients with sepsis and controls at T1 and T4 regarding mitoVO_2_ (Fig. 2C) and mitoDO_2_ (Fig. 2E). Correspondingly, we found no significant longitudinal changes between T1 and T4 (Fig. 2D and F).

### Prognostic value of PpIX-TSLT variables for 28-day and 180-day mortality

Of patients with a PpIX-TSLT measurement at T1, 15.8% (21/133) died within 28 days and 28.2% (37/131, *n* = 2 censored) died within 180 days after sepsis onset.

MitoDO_2_ was significantly lower in patients who deceased compared to those who survived (28 day mortality: 3.7 ± 1.9 *vs* 5 ± 2.7 mmHg/s; *p* = 0.011, *d* = − 0.50; 180 day mortality: 3.9 ± 2.1 vs 5.1 ± 2.8 mmHg/s; *p* = 0.010; *d* = − 0.46). MitoPO_2_ and mitoVO_2_ did not differ according to 28 day and 180-day mortality status (Supplementary Table 4). However, after adjusting for age, sex, comorbidities (Charlson comorbidity index), and disease severity (SOFA score), we found no association of PpIX-TSLT variables with 28 day and 180-day mortality (Supplementary Table 5).

### PpIX-TSLT variables: associative analyses

We present the complete associative analyses in Supplementary Tables 6–9. Here we report the results with *p* values < 0.05.

At T1, lower mitoDO_2_ was correlated with higher SOFA scores (*ρ* = − 0.23, *p* = 0.009, *n* = 133) and higher blood lactate concentration (*ρ* = − 0.21, *p* = 0.021, *n* = 126). Patients receiving vasopressor therapy on the day of T1 (4.3 ± 2.6 mmHg/s, *n* = 90) had significantly lower mitoDO_2_ compared to those without, with a small to medium effect size (*n* = 43, 5.6 ± 2.6 mmHg/s; *p* = 0.009; *d* = − 0.50; Supplementary Table 7). A lower periphery oxygen saturation was associated with a lower mitoPO_2_ (*ρ* = 0.18, *p* = 0.044, *n* = 131). Higher age was associated with a lower mitoVO_2_ (*ρ* = − 0.19, *p* = 0.031, *n* = 133).

Female patients had higher mitoPO_2_ than males with small to medium effect sizes (63.8 ± 19.1 *vs* 55.5 ± 18.7 mmHg; *p* = 0.017; *d* = 0.45). MitoDO_2_ tended to higher values in female patients compared to males (5.3 ± 2.9 *vs* 4.4 ± 2.4 mmHg/s; *p* = 0.078; *d* = 0.34; Supplementary Table 8). Given the observed differences, we conducted a more detailed analysis of the group comparison between patients at T1 and T4 and controls (Supplementary Table 8). By stratifying the cohort by sex, we confirmed the robustness of the finding that mitoPO_2_ was significantly lower in patients at T1 than in controls, across both male (*n* = 86 *vs* *n* = 50; 55.5 ± 18.7 *vs* 63.6 ± 17.8 mmHg; *p* = 0.013; *d* = − 0.44) and female subgroups (*n* = 47 *vs* *n* = 29; 63.8 ± 19.1 *vs* 73.8 ± 15.9 mmHg; *p* = 0.017; *d* = − 0.55). Longitudinal analysis revealed a significant increase of mitoPO_2_ between T1 and T4 in male patients (*n* = 24; 53.4 ± 19.5 *vs* 69.3 ± 21.9 mmHg; *p* = 0.011; *d* = − 0.77), but not in females (*n* = 16; 62.6 ± 16.3 *vs* 68.1 ± 20.1 mmHg; *p* = 0.296).

Higher sensor temperature was correlated with lower mitoPO_2_ in patients at T1 (*ρ* = − 0.24, *p* = 0.005, *n* = 133) and in controls (*ρ* = − 0.23, *p* = 0.038, *n* = 79). Higher body temperature was associated with higher mitoDO_2_ in controls (*ρ* = 0.27, *p* = 0.024, *n* = 71) but not in patients at T1 (*ρ* = 0.06, *p* = 0.478).

None of the other variables under consideration (mentioned in secondary endpoints) showed significant associations.

## Discussion

In this study, cellular oxygen metabolism was measured non-invasively in patients with sepsis in the acute and long-term course of the disease. During the acute phase of sepsis, mitoPO_2_ was significantly lower compared to controls, and it increased to the level of controls six months after onset. MitoDO_2_ was correlated with disease severity. For a classification of the PpIX-TSLT variables in the context of previous studies and discussion of the associations to parameters of the measurement environment, see Supplementary Discussion (Supplementary Material).

### PpIX-TSLT variables in the acute and long-term course of sepsis

Although there is increasing evidence for mitochondrial dysfunction in sepsis [7, 8, 10], it has not yet been clearly established whether primary mitochondrial dysfunction causes life-threatening organ dysfunction or whether limited tissue oxygenation leads to impaired mitochondrial function.

#### Mitochondrial oxygen tension (mitoPO_2_)

In the acute phase of sepsis, mitoPO_2_ was significantly decreased compared to healthy controls. Interestingly, mitoPO_2_ was equivalent to the controls, six months after sepsis onset. Lower muscle oxygen saturation index in patients with sepsis was demonstrated non-invasively using near-infrared spectroscopy compared to healthy [27]. The COMET system was used in an endotoxin model in both rats and healthy volunteers and showed a reduced mitoPO_2_ compared to controls [11, 23, 28]. The results of this study are in line with preliminary, preclinical work but require confirmation in larger multicentre studies.

#### Mitochondrial oxygen delivery (mitoDO_2_)

Tissue oxygenation and mitoDO_2_ are particularly dependent on adequate microcirculation. Reduced mitoDO_2_ was therefore considered to indicate impaired microcirculation [15]. The mitoDO_2_ of patients with sepsis did not differ from that of controls. MitoDO_2_ levels were significantly lower in patients who deceased within both the 28 day and 180-day periods after sepsis onset compared to those who survived. However, after adjusting for confounding variables including age, sex, comorbidities, and disease severity, this association was no longer statistically significant. This suggests that the initial observed association might be influenced by these confounding factors and indicates the need for further investigation into the role of mitoDO_2_ as a potential biomarker for patient outcomes. Reduced mitoDO_2_ was associated with disease severity. Supporting these results, microvascular changes in sepsis and their association with disease severity have been demonstrated non-invasively [4–6]. Furthermore, a correlation of altered macroscopic capillary refill time and mottling score with blood lactate concentration [29] and with SOFA score [30] has been described. Irrespective of sepsis, clinical studies have shown correlations between catecholamine therapy and reduced microcirculatory blood flow [31]. Thus, reduced mitoDO_2_ in patients receiving vasopressor therapy including catecholamines may be due to the vasoconstrictive effect of these drugs in addition to disease severity. However, we could show that impaired oxygen delivery at the mitochondrial level—not just changes in microcirculation—is associated with severity of sepsis.

#### Mitochondrial oxygen consumption (mitoVO_2_)

This study could not demonstrate a change in mitoVO_2_ in sepsis. The literature on this topic is heterogeneous [7–9, 32–36]. Using the COMET system, a decreased mitoVO_2_ was found in a rat sepsis model [28, 37]. Werfers Bettink and colleagues administered lipopolysaccharide to healthy volunteers to induce systemic inflammation and did not observe any changes in mitoVO_2_ [23]. Previous studies suggest changes in mitochondrial function in the sense of a threshold [20, 38]. In a rat sepsis model, time-dependent changes of hepatic mitochondrial respiration with normalization after 96 h have been shown [39]. The first measurement in patients was performed 3 ± 1 days after sepsis onset. Possible short-term, spontaneously reversible changes may not have been detected. Furthermore, there was heterogeneity in the cohort with regard to disease severity at T1, which is unavoidable due to the study design and its defined measurement time points. In future studies, it would be beneficial to compare measurements of mitoVO_2_ with ex vivo measurements of mitochondrial functions, such as high-resolution respirometry [40], in order to further investigate the mitochondrial function in sepsis.

The reduced level of mitochondrial oxygen (mitoPO_2_) in the acute phase of sepsis and the association of lower mitochondrial oxygen delivery (mitoDO_2_) with disease severity tend to indicate a restriction of oxygen supply without an impairment of mitochondrial oxygen consumption (mitoVO_2_) in the acute phase of sepsis.

### Strengths and limitations

The strict inclusion and exclusion criteria of the ICROS clinical study (e.g. excluding patients with severe pre-existing heart, kidney or liver disease, [25]) resulted in a specific cohort of patients with sepsis. The study was monocentric, and the cohort of those having PpIX-TSLT measurement at T1 and T4 was relatively small (*n* = 40 patients).

Important technical limitations include the limited diffusibility of ALA in the skin [5, 41] and the limited penetration of the sensor laser, which allows only the outer epidermis to be examined [12]. The skin may not fully reflect the status of central organ systems, and the influence of increased microcirculatory heterogeneity in the context of sepsis needs to be considered. To mitigate the impact of peripheral vasoconstriction and temperature fluctuations, the measurement was taken in the clavipectoral triangle. This study did not include a clinical assessment of cutaneous microcirculation parameters, such as Capillary Refill Time and Mottling score. Future research should combine PpIX-TSLT measurements with these assessments to better understand the effects of impaired microcirculation on cellular oxygen metabolism during sepsis. The values obtained for mitoPO_2_ using the PpIX-TSLT are higher than those reported in early studies, which ranged from less than 1 mmHg to 10 mmHg. It has been demonstrated that the cellular oxygen tension is more closely aligned with vascular oxygen levels [42, 43] and tissue oxygen tension [44]. The values of mitoPO₂ obtained through the COMET system are within the range of previously reported transcutaneous PO₂ values [45]. The cellular and tissue oxygen tension is understood to represent the regional equilibrium between oxygen consumption and supply [44]. The calculation of oxygen consumption (mitoVO_2_) and delivery (mitoDO_2_) is based on desaturation and resaturation measurements, respectively. It is estimated that over 90% of oxygen is metabolized within the mitochondria; however, extramitochondrial oxygen consumption may be a confounding factor. Antibiotic therapy is a fundamental component of sepsis treatment and was provided to all patients in this study, potentially affecting mitochondrial function due to the structural similarity between mitochondria and bacteria [46].

Despite the standardized measurement and analysis protocol, the PpIX-TSLT variables showed a high inter-individual variance as seen in previous studies of healthy subjects [15, 24, 38]. Furthermore, PpIX-TSLT variables were associated with the temperature of the COMET sensor and the exposure time of ALA prior to the measurement. In future studies, it is of particular importance to standardize the measurement protocol across study groups and to generate age- and sex-specific normative values. Prior to the implementation of the COMET system in individualized diagnostics, it is essential to further analyse potential covariates and technical aspects of the COMET system to reduce the inter- and intra-individual variability of measured values.

For secondary endpoints, p-values were not adjusted for multiple testing. The analysis of the data was exploratory. Therefore, our results need to be confirmed in larger studies.

## Conclusion

Non-invasive in vivo assessment of cellular oxygen metabolism in patients with sepsis revealed a reversible restriction of mitochondrial oxygen tension (mitoPO_2_) in the acute phase of sepsis. These results suggest that cellular oxygen metabolism in sepsis is characterized by a restriction of oxygen supply (hypoxia) without an impairment of mitochondrial oxygen utilization (mitoVO_2_). It therefore seems beneficial to pursue further development of the COMET system in order to facilitate the use of PpIX-TSLT in the individual assessment of patients with sepsis.

## Prior presentations

Parts of these findings were presented at the annual congress of the German Society for Anaesthesiology and Intensive Care Medicine (DGAI) in September 2024.

## Supplementary Information

Supplementary Material 1.

## Data Availability

The datasets used and/or analysed during the current study are available from the corresponding author on reasonable request.

## Abbreviations

95%-CI: 95% confidence interval
ALA: 5-aminolevulinic acid
COMET: Cellular oxygen METabolism
ICU: Intensive care unit
IQR: Interquartile range
mitoPO2: Mitochondrial oxygen consumption
mitoDO2: Mitochondrial oxygen delivery
mitoPO2: Mitochondrial oxygen tension
OR: Odds ratio
PpIX: Protoporphyrin IX
PpIX-TSLT: Protoporphyrin IX-triplet state lifetime technique
SD: Standard deviation
SOFA score: Sequential organ failure assessment score

## Time points

T0: Study enrolment
T1: 3 ± 1 days after sepsis onset
T4: 6 ± 2 months after sepsis onset
