# Supplementary material for: Reversible impairment of non-invasively assessed mitochondrial oxygen metabolism in the long-term course of patients with sepsis: a prospective monocentric cohort study
Source: Intensive Care Med Exp. 2025 Nov 14;13:114. doi: 10.1186/s40635-025-00808-x (PMC12615860; doi:10.1186/s40635-025-00808-x)
Supplement: Supplementary file 1 — Supplementary Material 1. [file 40635_2025_808_MOESM1_ESM.docx]

**Reversible impairment of non-invasively assessed mitochondrial oxygen metabolism in the long-term course of patients with sepsis: A prospective monocentric cohort study**

Anne Standke^1^, Charles Neu MD^1^, Philipp Baumbach PhD^1^, Alina K. Plooij MD^1^, Kornel Skitek^1^, Juliane Götze MD^1^, Sina M. Coldewey MD, PhD^2,1^

^1^Department of Anaesthesiology and Intensive Care Medicine, Septomics Research Centre, Translational Septomics, Jena University Hospital, Friedrich-Schiller-University Jena, Jena, Germany

^2^Department of Anaesthesiology and Perioperative Medicine, University Hospital Zürich, University of Zürich, Zürich, Switzerland

**Corresponding author:** Prof. Sina M. Coldewey MD, PhD, Department of Anaesthesiology and Perioperative Medicine, University Hospital Zürich, Rämistrasse 100, 8006 Zürich, Switzerland. Tel.: +41 44 255 26 95. E-mail: sina.coldewey@uzh.ch

**Conflict of Interests:** The authors declare no competing interests.

**Clinical trial registration:** NCT03620409 (Ethics vote: 5276-09/17; German Register of Clinical Studies: DRKS00013347), Principal investigator: Sina M. Coldewey, Date of Registration: 11-30-2017

**Prior presentations:** Parts of these findings were presented at the annual congress of the German Society for Anaesthesiology and Intensive Care Medicine (DGAI) in September 2024.

CONTENT

[1 Supplementary Methods 3](#_Toc205566818)

[1.1 Inclusion and Exclusion criteria 3](#_Toc205566819)

[2 Tables 3](#_Toc205566820)

[3 Figures 10](#_Toc205566821)

[4 Supplementary Discussion 11](#_Toc205566822)

[4.1 Classification of the PpIX-TSLT variables in context of previous studies 11](#_Toc205566823)

[4.2 Analysis of potential covariates of the PpIX-TSLT variables 12](#_Toc205566824)

[5 References 15](#_Toc205566825)

# Supplementary Methods

## Inclusion and Exclusion criteria

The main exclusion criteria of the ICROS study were significant cardiac or pulmonary disease, Child C liver cirrhosis, end-stage chronic kidney disease on dialysis, and remaining life expectancy < 6 months due to pre-existing conditions (see full list [1]). The COMET measurement was contraindicated in patients with allergy to contents of the Alacare® patch (Photonamic, Wedel, Germany), porphyria, and skin diseases aggravated by sunlight, or increased sensitivity to light.

# Tables

Table 1 Descriptive statistic and group comparison of PpIX-TSLT variables of patients with sepsis and controls. Mean and standard deviation (SD) of mitochondrial oxygen tension (mitoPO_2_),
-consumption (mitoVO_2_) and -delivery (mitoDO_2_) and Bonferroni-Holm adjusted p values of two-sided t-test for independent samples (p) of the comparisons of the patients (3±1 days (T1) and 6±2 months after onset (T4)) to controls and Cohen’s d (d) are shown. P<0.05 are printed in bold.

|  | Controls  (n=79) |  | Patients | | | | | | |
| --- | --- | --- | --- | --- | --- | --- | --- | --- | --- |
|  |  |  | T1 (n=133) | | |  | T4 (n=43) | | |
| Variables | mean ± SD |  | mean ± SD | p ^vs. controls^ | d |  | mean ± SD | p ^vs. controls^ | d |
| mitoPO_2_ [mmHg] | 67.3 ± 17.7 |  | 58.4 ± 19.2 | **0.002** | 0.48 |  | 67.9 ± 20.7 | 0.994 | −0.03 |
| mitoVO_2_ [mmHg/s] | 3.6 ± 1.6 |  | 3.4 ± 1.8 | >0.999 | 0.06 |  | 3.9 ± 2.7 | 0.994 | −0.15 |
| mitoDO_2_ [mmHg/s] | 4.8 ± 20 |  | 4.7 ± 2.6 | >0.999 | 0.01 |  | 5.5 ± 3.0 | 0.494 | −0.30 |

*PpIX-TSLT, Protoporphyrin IX-triplet state lifetime technique*

Table 2 Descriptive statistic and longitudinal comparison of PpIX-TSLT variables of patients with sepsis. Mean and standard deviation (SD) of mitochondrial oxygen tension (mitoPO_2_), -consumption (mitoVO_2_) and -delivery (mitoDO_2_) and p values of two-sided t-test for independent samples (p) of the comparisons of the patients (3±1 days (T1) and 6±2 months after onset (T4)) to controls and p values of two-sided t-test for paired samples of the comparisons of T1 to T4 and Cohen’s d (d) are shown. P<0.05 are printed in bold.

|  | Controls (n=79) |  | Patients | | | | | | | | |
| --- | --- | --- | --- | --- | --- | --- | --- | --- | --- | --- | --- |
|  |  |  | T1 (n=40) | | |  | T4 (n=40) | | | | |
| Variables | mean ± SD |  | mean ± SD | p ^vs. controls^ | d |  | mean ± SD | p ^vs. controls^ | d | p ^T1vs.T4^ | d |
| mitoPO_2_ [mmHg] | 67.3 ± 17.7 |  | 57.1 ± 18.7 | **0.005** | 0.57 |  | 68.8 ± 21.0 | 0.704 | −0.08 | **0.006** | −0.46 |
| mitoVO_2_ [mmHg/s] | 3.6 ± 1.6 |  | 3.8 ± 2.1 | 0.561 | −0.12 |  | 3.8 ± 2.6 | 0.527 | −0.14 | 0.903 | −0.02 |
| mitoDO_2_ [mmHg/s] | 4.8 ± 2.0 |  | 4.9 ± 3 | 0.806 | −0.05 |  | 5.0 ± 2.4 | 0.634 | −0.10 | 0.880 | −0.02 |

*PpIX-TSLT, Protoporphyrin IX-triplet state lifetime technique*

Table 3 Drop-out Analysis. Comparison of the PpIX-TSLT variables of acute phase of sepsis (3 ± 1 days after onset, T1) between patients with a measurement in the long-term course (6 ± 2 months after onset, T4) and those without. Mean and standard deviation of mitochondrial oxygen tension (mitoPO_2_), -consumption (mitoVO_2_) and -delivery (mitoDO_2_) and p values of two-sided t-test for independent samples (p) and Cohen’s d (d) are shown. P<0.05 are printed in bold.

|  | Patients with T1 (n=133) | |  |  |
| --- | --- | --- | --- | --- |
| Variables | No T4 (n=93) | T1 and T4 (n=40) | p | d |
| mitoPO_2_ [mmHg] | 59.0 ± 19.5 | 57.1 ± 18.7 | 0.592 | 0.10 |
| mitoVO_2_ [mmHg/s] | 3.3 ± 1.7 | 3.8 ± 2.1 | 0.213 | −0.26 |
| mitoDO_2_ [mmHg/s] | 4.7 ± 2.5 | 4.9 ± 3 | 0.706 | −0.08 |

*PpIX-TSLT, Protoporphyrin IX-triplet state lifetime technique*

Table 4 PpIX-TSLT variables of acute phase of sepsis (3 ± 1 days after onset, T1) grouped according to 28-day and 180-day mortality. Mean and standard deviation of mitochondrial oxygen tension (mitoPO_2_), -consumption (mitoVO_2_) and -delivery (mitoDO_2_) and p values of two-sided t-test for independent samples (p) and Cohen’s d (d) are shown. P<0.05 are printed in bold.

|  | 28-day mortality (n=133) | | | |  | 180-day mortality (n=131), n=2 censored | | | |
| --- | --- | --- | --- | --- | --- | --- | --- | --- | --- |
| Variables | Deceased (n=21) | Survived (n=112) | p | d |  | Deceased (n=37) | Survived (n=94) | p | d |
| mitoPO_2_ [mmHg] | 57.3 ± 17.1 | 58.6 ± 19.6 | 0.748 | −0.07 |  | 58.6 ± 18.5 | 58.1 ± 19.6 | 0.889 | 0.03 |
| mitoVO_2_ [mmHg/s] | 3.1 ± 1.4 | 3.5 ± 1.9 | 0.277 | −0.21 |  | 3.0 ± 1.3 | 3.6 ± 1.9 | 0.059 | −0.32 |
| mitoDO_2_ [mmHg/s] | 3.7 ± 1.9 | 5 ± 2.7 | **0.011** | −0.50 |  | 3.9 ± 2.1 | 5.1 ± 2.8 | **0.010** | −0.46 |

*PpIX-TSLT, Protoporphyrin IX-triplet state lifetime technique*

Table 5 **Prognostic value of** PpIX-TSLT **variables** of acute phase of sepsis (3 ± 1 days after onset, T1) **for 28-day and 180-day mortality.** Adjusted Odds ratio, corresponding 95%-confidence interval (95%CI) and p value (p) of a logistic regression are shown. Mortality was modelled as dependent variable, mitochondrial oxygen tension (mitoPO_2_), -consumption (mitoVO_2_) and -delivery (mitoDO_2_) were modelled separately as independent variables. The data on Sequential organ failure assessment (SOFA) score at T1, Charlson comorbidity index (CCI), sex, and age relate to the models with mitoDO_2_ as independent variable. An odds ratio < 1 indicates a decreased, an odds ratio > 1 indicates an increased mortality risk. All continuous variables were z-standardized before modelling. P<0.05 are shown in bold.

|  |  | Variable | Odds ratio | [95%CI] | p |
| --- | --- | --- | --- | --- | --- |
| 28-day mortality | survived : deceased  (n=112 : 21) | mitoPO_2_ | 1.05 | [0.57-1.93] | 0.864 |
|  |  | mitoVO_2_ | 0.88 | [0.45-1.54] | 0.674 |
|  |  | mitoDO_2_ | 0.63 | [0.31-1.16] | 0.174 |
|  |  | SOFA | 3.28 | [1.81-6.62] | **<0.001** |
|  |  | CCI | 1.04 | [0.61-1.69] | 0.880 |
|  |  | male sex | 0.63 | [0.21-1.90] | 0.407 |
|  |  | age | 1.80 | [0.94-3.91] | 0.106 |
| 180-day mortality | survived : deceased  (n=94 : 37) | mitoPO_2_ | 1.05 | [0.65-1.69] | 0.829 |
|  |  | mitoVO_2_ | 0.72 | [0.42-1.17] | 0.213 |
|  |  | mitoDO_2_ | 0.69 | [0.41-1.09] | 0.130 |
|  |  | SOFA | 2.66 | [1.65-4.52] | **<0.001** |
|  |  | CCI | 1.66 | [1.09-2.59] | **0.021** |
|  |  | male sex | 0.72 | [0.29-1.79] | 0.471 |
|  |  | age | 1.14 | [0.72-1.86] | 0.590 |

*PpIX-TSLT, Protoporphyrin IX-triplet state lifetime technique*

Table 6 Descriptive statistics of clinical parameters and analysis of correlation with PpIX-TSLT variables. Median, interquartile range [IQR] of the clinical parameters and Spearman’s rank correlation coefficient (ρ) with the corresponding p-value (p) are shown for mitochondrial oxygen tension (mitoPO_2_), -consumption (mitoVO_2_) and -delivery (mitoDO_2_), demographic and clinical variables and laboratory parameters of all patients with a PpIX-TSLT measurement in the acute course of the disease (3 ± 1 days after sepsis diagnosis; n=133) and the duration of therapy for those patients who received ICU therapy at T1 and could be discharged from hospital (n=75). P<0.05 are shown in bold.

|  | Variable |  | |  | Correlative analyses | | | | | | | | |
| --- | --- | --- | --- | --- | --- | --- | --- | --- | --- | --- | --- | --- | --- |
|  |  | Descriptive statistics | |  | mitoPO_2_ | |  | mitoVO_2_ | |  | mitoDO_2_ | | |
|  |  | n | median [IQR] |  | ρ | p |  | ρ | p |  | ρ | p | |
| Demo-graphy | Age [years] | 133 | 66 [56, 75] |  | −0.07 | 0.406 |  | −0.19 | **0.031** |  | −0.16 | 0.059 | |
|  | BMI [kg/m²] | 133 | 27.9 [23.8, 32.4] |  | −0.08 | 0.349 |  | −0.02 | 0.795 |  | <0.01 | 0.984 | |
| Clinical Variables | SOFA [points] | 133 | 8 [5, 10] |  | −0.10 | 0.270 |  | −0.01 | 0.949 |  | −0.23 | **0.009** | |
|  | Heart rate [bpm] | 129 | 80 [71, 95] |  | <0.01 | 0.986 |  | 0.07 | 0.421 |  | −0.11 | 0.197 | |
|  | Systolic blood pressure [mmHg] | 129 | 126 [112, 143] |  | −0.01 | 0.954 |  | 0.08 | 0.375 |  | 0.14 | 0.109 | |
|  | Diastolic blood pressure [mmHg] | 129 | 60 [52, 66] |  | −0.09 | 0.320 |  | −0.02 | 0.862 |  | 0.12 | 0.159 | |
|  | SpO_2_ (%) | 131 | 97 [95, 98] |  | 0.18 | **0.044** |  | 0.01 | 0.890 |  | 0.15 | 0.096 | |
| Laboratory parameters | Lactate [mmol/l] | 126 | 1.4 [1.1, 1.9] |  | 0.04 | 0.648 |  | 0.13 | 0.158 |  | −0.21 | **0.021** | |
|  | Bilirubin [µmol/l] | 129 | 13.0 [6.0, 26.0] |  | −0.07 | 0.448 |  | −0.02 | 0.851 |  | −0.05 | 0.551 | |
|  | Hemoglobin  [mmol/l] | 132 | 5.2 [4.9, 5.9] |  | −0.08 | 0.347 |  | −0.03 | 0.720 |  | 0.02 | 0.808 | |
| Duration of  therapy | LOS in hospital [d] | 75 | 30 [23, 49] |  | 0.06 | 0.580 |  | 0.07 | 0.555 |  | −0.03 | 0.817 | |
|  | LOS at ICU [d] | 75 | 17 [8, 26] |  | −0.05 | 0.642 |  | 0.09 | 0.432 |  | −0.16 | 0.166 | |
|  | Vasopressor therapy [d] | 72 | 11 [5, 18] |  | −0.10 | 0.406 |  | 0.03 | 0.792 |  | −0.17 | 0.149 | |
|  | BMI: Body Mass Index; SOFA: Sequential Organ Assessment Score; SpO_2_: oxygen saturation; LOS: length of stay | | | | | | | | | | | |  |

*PpIX-TSLT, Protoporphyrin IX-triplet state lifetime technique*

Table 7 PpIX-TSLT variables of acute phase of sepsis (3 ± 1 days after onset, T1), grouped according to catecholamine therapy on the day of measurement. Mean and standard deviation of mitochondrial oxygen tension (mitoPO_2_), -consumption (mitoVO_2_) and -delivery (mitoDO_2_) and p-values of independent samples t-test (p) and Cohen's d (d) are shown. P < 0.05 are shown in bold.

|  | Catecholamine therapy | |  |  |
| --- | --- | --- | --- | --- |
| Variables | Yes^†^ (n=90) | No (n=43) | p | d |
| mitoPO_2_ [mmHg] | 57.6 ± 18.8 | 60.2 ± 20.1 | 0.475 | −0.14 |
| mitoVO_2_ [mmHg/s] | 3.4 ± 1.8 | 3.5 ± 1.9 | 0.915 | −0.02 |
| mitoDO_2_ [mmHg/s] | 4.3 ± 2.6 | 5.6 ± 2.6 | **0.009** | −0.50 |

*PpIX-TSLT, Protoporphyrin IX-triplet state lifetime technique*

*^†^ n=85 with noradrenalin (only), n=3 noradrenalin and vasopressin, n=2 noradrenalin with dobutamine*

Table 8 PpIX-TSLT variables of acute phase of sepsis (3 ± 1 days after onset, T1) grouped according to sex. Mean and standard deviation of mitochondrial oxygen tension (mitoPO_2_), -consumption (mitoVO_2_) and -delivery (mitoDO_2_) and p values of two-sided t-test for independent samples of the comparisons of the patients (3±1 days (T1) and 6±2 months after onset (T4)) to controls (p_vs. controls_) and of females to males (p_female vs. male_) and Cohen’s d (d) are shown. P values of two-sided t-test for paired samples are shown for longitudinal comparisons of T1 to T4 (p_T1 vs. T4_). P<0.05 are printed in bold.

|  |  | Variables | n |  | p ^female vs. male^ | \|d\| | p ^vs. controls^ | \|d\| | p ^T1 vs.T4^ | \|d\| |
| --- | --- | --- | --- | --- | --- | --- | --- | --- | --- | --- |
| Healthy | Female | mitoPO_2_  [mmHg] | 29 | 73.8 ± 15.9 |  |  |  |  |  |  |
|  | Male |  | 50 | 63.6 ± 17.8 | **0.011** | 0.59 |  |  |  |  |
|  | Female | mitoVO_2_ [mmHg/s] | 29 | 3.4 ± 1.7 |  |  |  |  |  |  |
|  | Male |  | 50 | 3.7 ± 1.6 | 0.433 | 0.19 |  |  |  |  |
|  | Female | mitoDO_2_  [mmHg/s] | 29 | 5.2 ± 2.0 |  |  |  |  |  |  |
|  | Male |  | 50 | 4.5 ± 2.0 | 0.160 | 0.33 |  |  |  |  |
| Group comparison | T1 female | mitoPO_2_  [mmHg] | 47 | 63.8 ± 19.1 |  |  | **0.017** | 0.55 |  |  |
|  | T1 male |  | 86 | 55.5 ± 18.7 | **0.017** | 0.45 | **0.013** | 0.44 |  |  |
|  | T1 female | mitoVO_2_  [mmHg/s] | 47 | 3.2 ± 1.5 |  |  | 0.637 | 0.12 |  |  |
|  | T1 male |  | 86 | 3.6 ± 2.0 | 0.175 | 0.23 | 0.807 | 0.04 |  |  |
|  | T1 female | mitoDO_2_  [mmHg/s] | 47 | 5.3 ± 2.9 |  |  | 0.813 | 0.05 |  |  |
|  | T1 male |  | 86 | 4.4 ± 2.4 | 0.078 | 0.34 | 0.836 | 0.04 |  |  |
|  | T4 female | mitoPO_2_ [mmHg] | 16 | 68.1 ± 20.1 |  |  | 0.342 | 0.32 |  |  |
|  | T4 male |  | 27 | 67.8 ± 21.4 | 0.964 | 0.01 | 0.388 | 0.22 |  |  |
|  | T4 female | mitoVO_2_ [mmHg/s] | 16 | 3.9 ± 3.6 |  |  | 0.596 | 0.20 |  |  |
|  | T4 male |  | 27 | 3.9 ± 2.1 | 0.989 | 0.01 | 0.682 | 0.11 |  |  |
|  | T4 female | mitoDO_2_  [mmHg/s] | 16 | 5.4 ± 2.8 |  |  | 0.833 | 0.07 |  |  |
|  | T4 male |  | 27 | 5.6 ± 3.2 | 0.825 | 0.07 | 0.133 | 0.42 |  |  |
| Longitudinal comparison | T1 female | mitoPO_2_  [mmHg] | 16 | 62.6 ± 16.3 |  |  |  |  |  |  |
|  | T1 male |  | 24 | 53.4 ± 19.5 |  |  |  |  |  |  |
|  | T1 female | mitoVO_2_  [mmHg/s] | 16 | 2.7 ± 1.1 |  |  |  |  |  |  |
|  | T1 male |  | 24 | 4.5 ± 2.4 |  |  |  |  |  |  |
|  | T1 female | mitoDO_2_  [mmHg/s] | 16 | 6.4 ± 3.0 |  |  |  |  |  |  |
|  | T1 male |  | 24 | 3.9 ± 2.5 |  |  |  |  |  |  |
|  | T4 female | mitoPO_2_ [mmHg] | 16 | 68.1 ± 20.1 |  |  |  |  | 0.296 | 0.30 |
|  | T4 male |  | 24 | 69.3 ± 21.9 |  |  |  |  | **0.011** | 0.77 |
|  | T4 female | mitoVO_2_ [mmHg/s] | 16 | 3.9 ± 3.6 |  |  |  |  | 0.220 | 0.44 |
|  | T4 male |  | 24 | 3.8 ± 1.7 |  |  |  |  | 0.192 | 0.33 |
|  | T4 female | mitoDO_2_  [mmHg/s] | 16 | 5.4 ± 2.8 |  |  |  |  | 0.267 | 0.37 |
|  | T4 male |  | 24 | 4.7 ± 2.1 |  |  |  |  | 0.160 | 0.37 |

*PpIX-TSLT, Protoporphyrin IX-triplet state lifetime technique*

Table 9 Descriptive statistics for parameters of the measurement environment and analyses of correlation to the PpIX-TSLT variables of controls and patients in the acute phase of sepsis (3 ± 1 days after onset, T1). Spearman’s rank correlation coefficients (ρ) with the corresponding p-values (p) are shown. P<0.05 are shown in bold.

|  |  |  |  |  | Correlative analyses | | | | | | | |
| --- | --- | --- | --- | --- | --- | --- | --- | --- | --- | --- | --- | --- |
|  | Variables of the measurement environment | Descriptive statistics | |  | mitoPO_2_ | |  | mitoVO_2_ | |  | mitoDO_2_ | |
|  |  | n | Median [IQR] |  | ρ | p |  | ρ | p |  | ρ | p |
| Controls (n=79) | Duration of ALA application [h] | 79 | 11.3 [10, 12.1] |  | −0.05 | 0.636 |  | 0.05 | 0.650 |  | −0.04 | 0.699 |
|  | Sensor temperature [°C] | 79 | 30.5 [29.9, 31.2] |  | −0.23 | **0.038** |  | 0.10 | 0.391 |  | −0.17 | 0.127 |
|  | Skin temperature [°C] | 77 | 32.6 [31.6, 33.5] |  | 0.12 | 0.292 |  | 0.17 | 0.141 |  | 0.16 | 0.170 |
|  | Body temperature [°C] | 71 | 36.5 [36.2, 36.7] |  | 0.16 | 0.190 |  | 0.07 | 0.575 |  | 0.27 | **0.024** |
|  | Room temperature [°C] | 79 | 23.0 [22.4, 23.7] |  | 0.11 | 0.332 |  | −0.04 | 0.714 |  | 0.03 | 0.823 |
| Patients at T1 (n=134) | Duration of ALA application [h] | 133 | 6.9 [6.3, 7.7] |  | 0.07 | 0.417 |  | 0.16 | 0.068 |  | −0.14 | 0.115 |
|  | Sensor temperature [°C] | 133 | 30.9 [30.1, 31.6] |  | −0.24 | **0.005** |  | −0.06 | 0.474 |  | 0.01 | 0.933 |
|  | Skin temperature [°C] | 118 | 33.8 [32.8, 34.4] |  | −0.10 | 0.282 |  | 0.09 | 0.352 |  | 0.12 | 0.211 |
|  | Body temperature [°C] | 123 | 37.1 [36.5, 37.7] |  | −0.12 | 0.202 |  | 0.08 | 0.357 |  | 0.06 | 0.478 |
|  | Room temperature [°C] | 130 | 23.1 [22.4, 24.0] |  | 0.08 | 0.359 |  | −0.04 | 0.640 |  | 0.11 | 0.195 |
|  | ALA: 5-aminolaevulinic acid; h: hours; IQR: interquartile range; mitoPO_2_: mitochondrial oxygen tension; mitoVO_2_: mitochondrial oxygen consumption; mitoDO_2_: mitochondrial oxygen delivery; n: number; PpIX-TSLT: Protoporphyrin IX-triplet state lifetime technique | | | | | | | | | | | |

# Figures

**
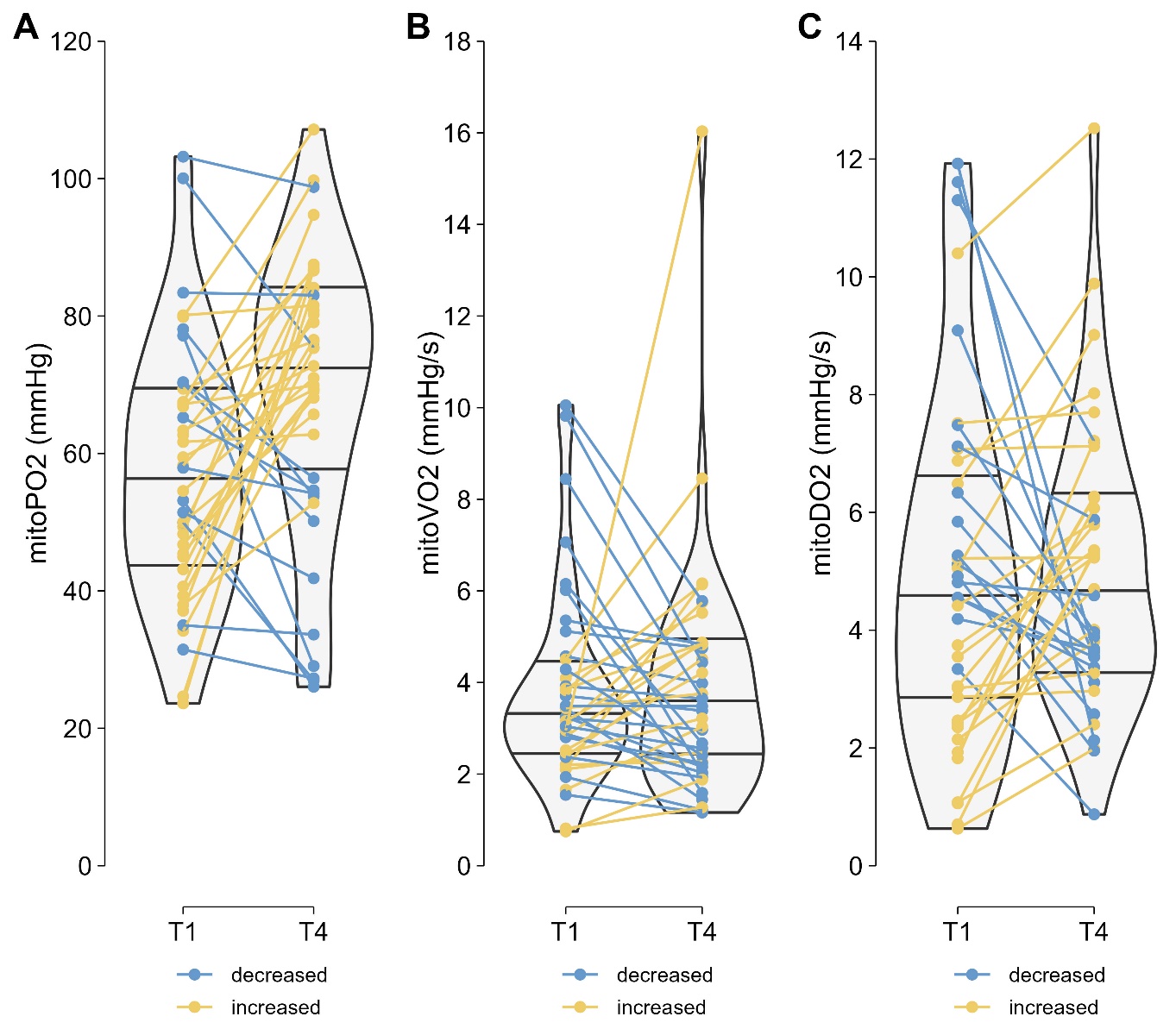
**

Supplementary Fig. 1 Individual changes in protoporphyrin IX-triplet state lifetime technique (PpIX-TSLT) variables. Lines connect individual values between T1 (3±1 days after sepsis onset) and T4 (6±2 months after sepsis onset) of (A) mitochondrial oxygen tension (mitoPO_2_), (B) mitochondrial oxygen consumption (mitoVO_2_), and (C) mitochondrial oxygen delivery (mitoDO_2_). The violin plots display the distribution of the data including median (middle line), first and third quartile (lower/upper line).

# Supplementary Discussion

## Classification of the PpIX-TSLT variables in context of previous studies

**Mitochondrial oxygen tension (mitoPO_2_).** The Protoporphyrin IX-triplet state lifetime technique (PpIX-TSLT) we employed has been validated both in pre-clinical studies and in healthy subjects. Harms and colleagues calibrated mitoPO_2_ measurements in rats by comparison with simultaneous measurements of the cutaneous microvascular oxygen tension [2]. Three different conditions were used: normal skin tissue, nonrespiration skin tissue due to the application of cyanide, and anoxic skin tissue after the ventilation with 100% nitrogen [2]. Ubbink and colleagues validated the measurement of mitoPO_2_ and mitoVO_2_ in a preliminary study of healthy subjects. To validate mitoPO_2_ they used the same approach as in vivo calibration in animals, i.e. to use a combination of blocking oxygen supply by microvascular occlusion and blocking mitochondrial respiration by cyanide cream. MitoVO_2_ measurements were validated compared to spectroscopic and transcutaneous techniques in microvascular occlusion measurements [3]. The mean±SD mitoPO_2_ within this cohort was 67.3±17.7 mmHg in controls, 58.4 ±19.2 mmHg in patients with sepsis at T1, and 67.9±20.7 mmHg at T4. Compared to available studies using the COMET system, mitoPO_2_ measured in this study is within the ranges of most of preclinical studies (mean values between 59–68 mmHg [4, 5]), controls (mean values between 63 mmHg and 72 mmHg, [3, 6-8]) and in patients before elective surgery (mean values between 58–70 mmHg, [9-12]). Compared to the feasibility study, mitoPO_2_ values were confirmed in this now expanded cohort [13]. In contrast, three other published pilot studies found mitoPO_2_ values that were on average about 20 mmHg lower (mean range: 44–51 mmHg, [6, 14, 15]) than in the controls and patients at T4 in this study. Technical differences in the prototype of the COMET system [6, 14] and demographic differences between the cohorts [15] may account for the observed discrepancy with these studies. The deviation of mitoPO_2_ found in this study from that of previous work is partly due to the sex distribution, as only young males were included in the pilot study [15].

**Mitochondrial oxygen consumption (mitoVO_2_)**. The mean±SD mitoVO_2_ within this cohort was 3.6±1.6 mmHg/s in controls, 3.4±1.8 mmHg/s in patients with sepsis at T1 and 3.9±2.7 mmHg/s at T4. MitoVO_2_ between 4.1 mmHg/s and 7.7 mmHg/s was found in the majority of the previously studied cohorts of healthy controls [6-8, 14-17] which is higher than in the present study. In contrast, Ubbink and colleagues found mitoVO_2_, substantially lower than the above studies (2.1 mmHg/s), and showed a correlation between selected measurement area and measured mitoVO_2_ [3]. The discrepancy in mitoVO_2_ may be due to the infraclavicular measurement site or to the different strategies to evaluate the raw COMET values used by different groups. MitoVO_2_ of septic patients at T1 was equivalent to that of the smaller cohort [13] and thus validated.

**Mitochondrial oxygen delivery (mitoDO_2_)**. The mean±SD mitoDO_2_ within this cohort was 4.8±2.0 mmHg/s in controls, 4.7±2.6 mmHg/s in patients with sepsis at T1 and 5.5±3.0 mmHg/s at T4. The variable of mitoDO_2_ was defined and described for the first time in 2019 by our working group [7]. So far, only a small amount of reference data is available from this working group. In the two pilot studies, the mean mitoDO_2_ (6.52 mmHg/s, [7] and 6.78 mmHg/s, [15]) was slightly higher than in controls of this study. This difference may be due to the different demographics of the cohorts and the different measurement area. The level of mitoDO_2_ found in the feasibility study in acute phase of sepsis could be confirmed in this larger cohort [13].

## Analysis of potential covariates of the PpIX-TSLT variables

**Sex-specific differences in PpIX-TSLT variables.** As previously described [13], significantly higher mitoPO_2_ and mitoDO_2_ were found in female patients. The disruption of the microcirculation due to endothelial dysfunction, triggered by oxidative stress, has been described as an important factor in the pathophysiology of sepsis [18]. Lower production of reactive oxygen species and a higher antioxidant activity in the mitochondria of heart and brain cells of female mice has been shown [19]. A link between the inflammatory response and estrogen has been described in a rat sepsis-model [20]. This underlines the need for further sex-specific research. Therefore, we conducted a more detailed analysis of the group comparison between patients and controls at T1 By stratifying the cohort by sex, we validated the robustness of the finding that mitoPO_2_ was significantly lower in patients at T1 compared to controls, consistent across both male and female subgroups. Longitudinal analysis demonstrated a significant increase in mitoPO_2_ between T1 and T4 in male patients; however, this was not observed in female patients. It is important to recognize that the cohort of female patients with measurements at both T1 and T4 was quite limited, comprising only 16 individuals, which likely affected the statistical power of the analysis.

**Mitochondrial oxygen tension (mitoPO_2_).** In both patients at T1 and controls, lower COMET system sensor temperature was associated with higher mitoPO_2_. The manufacturer of the COMET refers to this correlation in the device documentation. However, there is no device internal sensor temperature dependent adjustment. According to the internal working instructions of the working group, the measuring temperature of the sensor should be close to the physiological skin temperature at 32°C [21]. The mean sensor temperature was 31°C. Therefore, and due to the low correlation coefficient, no significant influence on the measured values can be assumed in this cohort.

**Mitochondrial oxygen consumption (mitoVO_2_)**. In patients with sepsis at T1, we identified as association of higher age with lower mitoVO_2_. The association between reduced mitochondrial function and the progression of age has been well-established in ex vivo studies [22-24]. A potential underlying mechanism is the accumulation of mutations within mitochondrial DNA that occurs with increasing chronological age [25, 26]. We did not find any associations between measuring environment parameters and mitoVO_2_ in either patients with sepsis or controls.

**Mitochondrial oxygen delivery (mitoDO_2_)**. Neu and colleagues identified an association of longer ALA exposure time, as a modifiable covariate, with lower mitoDO_2_ in a pilot study of patients with sepsis [13]. This association was neither confirmed in this lager cohort of patients with sepsis nor in controls. Due to the organizational necessity to perform the ambulatory measurements in the morning, the controls and patients at T4 were instructed to apply the patch on the evening before the examination. For the patients at T1, the ALA patch was applied under clinical conditions on the morning of the measurement day. This resulted in a significant difference in the ALA application time of the patients at T1 (7.5±2.3 h) compared to the healthy controls (11.3±2.6 h; p<0.001) and at T4 (12.1±3.2 h; p<0.001). However, after adjusting for ALA application time in regression models, the results for group comparisons at T1 were similar. In addition, ALA application time was only significantly associated with T1 mitoVO_2_ in the regression models (β_z_ = 0.25, 95%CI: 0.08–0.42, p=0.003). This suggests, a consistent ALA exposure time should be ensured.

# References

1. Coldewey SM, Neu C, Baumbach P, Scherag A, Goebel B, Ludewig K, Bloos F, Bauer M, (2020) Identification of cardiovascular and molecular prognostic factors for the medium-term and long-term outcomes of sepsis (ICROS): protocol for a prospective monocentric cohort study. BMJ Open 10: e036527. https//doi.org/10.1136/bmjopen-2019-036527

2. Harms FA, Bodmer SIA, Raat NJH, Stolker RJ, Mik EG, (2012) Validation of the protoporphyrin IX-triplet state lifetime technique for mitochondrial oxygen measurements in the skin. Opt Lett 37: 2625-2627. https//doi.org/10.1364/OL.37.002625

3. Ubbink R, Wefers Bettink MA, van Weteringen W, Mik EG, (2021) Mitochondrial oxygen monitoring with COMET: verification of calibration in man and comparison with vascular occlusion tests in healthy volunteers. Journal of Clinical Monitoring and Computing 35: 1357-1366. https//doi.org/10.1007/s10877-020-00602-y

4. Harms FA, Voorbeijtel WJ, Bodmer SI, Raat NJ, Mik EG, (2013) Cutaneous respirometry by dynamic measurement of mitochondrial oxygen tension for monitoring mitochondrial function in vivo. Mitochondrion 13: 507-514. https//doi.org/10.1016/j.mito.2012.10.005

5. Harms FA, Bodmer SIA, Raat NJH, Mik EG, (2015) Non-invasive monitoring of mitochondrial oxygenation and respiration in critical illness using a novel technique. Critical Care 19: 343. https//doi.org/10.1186/s13054-015-1056-9

6. Harms FA, Bodmer SIA, Raat NJH, Mik EG, (2015) Cutaneous mitochondrial respirometry: non-invasive monitoring of mitochondrial function. Journal of Clinical Monitoring and Computing 29: 509-519. https//doi.org/10.1007/s10877-014-9628-9

7. Baumbach P, Neu C, Derlien S, Bauer M, Nisser M, Buder A, Coldewey SM, (2019) A pilot study of exercise-induced changes in mitochondrial oxygen metabolism measured by a cellular oxygen metabolism monitor (PICOMET). Biochim Biophys Acta Mol Basis Dis 1865: 749-758. https//doi.org/10.1016/j.bbadis.2018.12.003

8. Streng L, de Wijs CJ, Raat NJH, Specht PAC, Sneiders D, van der Kaaij M, Endeman H, Mik EG, Harms FA, (2022) In Vivo and Ex Vivo Mitochondrial Function in COVID-19 Patients on the Intensive Care Unit. Biomedicines 1010.3390/biomedicines10071746

9. Costerus SA, Bettink MW, Tibboel D, de Graaff JC, Mik EG, (2020) Mitochondrial Oxygen Monitoring During Surgical Repair of Congenital Diaphragmatic Hernia or Esophageal Atresia: A Feasibility Study. Frontiers in Pediatrics 8

10. Harms FA, Brandt-Kerkhof ARM, Mik EG, (2021) Monitoring of mitochondrial oxygenation during perioperative blood loss. BMJ Case Reports 14: e237789. https//doi.org/10.1136/bcr-2020-237789

11. Harms FA, Ubbink R, de Wijs CJ, Ligtenberg MP, Ter Horst M, Mik EG, (2022) Mitochondrial Oxygenation During Cardiopulmonary Bypass: A Pilot Study. Front Med (Lausanne) 9: 785734. https//doi.org/10.3389/fmed.2022.785734

12. Harms FA, Streng L, Wefers Bettink MA, de Wijs CJ, Römers LH, Janse R, Stolker RJ, Mik EG, (2023) Monitoring of mitochondrial oxygen tension in the operating theatre: An observational study with the novel COMET® monitor. PLoS One 18: e0278561. https//doi.org/10.1371/journal.pone.0278561

13. Neu C, Baumbach P, Plooij AK, Skitek K, Gotze J, von Loeffelholz C, Schmidt-Winter C, Coldewey SM, (2020) Non-invasive Assessment of Mitochondrial Oxygen Metabolism in the Critically Ill Patient Using the Protoporphyrin IX-Triplet State Lifetime Technique-A Feasibility Study. Front Immunol 11: 757. https//doi.org/10.3389/fimmu.2020.00757

14. Harms FA, Stolker RJ, Mik EG, (2016) Cutaneous Respirometry as Novel Technique to Monitor Mitochondrial Function: A Feasibility Study in Healthy Volunteers. PLoS One 11: e0159544. https//doi.org/10.1371/journal.pone.0159544

15. Baumbach P, Schmidt-Winter C, Hoefer J, Derlien S, Best N, Herbsleb M, Coldewey SM, (2020) A Pilot Study on the Association of Mitochondrial Oxygen Metabolism and Gas Exchange During Cardiopulmonary Exercise Testing: Is There a Mitochondrial Threshold? Frontiers in medicine 7: 585462-585462. https//doi.org/10.3389/fmed.2020.585462

16. Ubbink R, Bettink MAW, Janse R, Harms FA, Johannes T, Münker FM, Mik EG, (2017) A monitor for Cellular Oxygen METabolism (COMET): monitoring tissue oxygenation at the mitochondrial level. J Clin Monit Comput 31: 1143-1150. https//doi.org/10.1007/s10877-016-9966-x

17. van Diemen MPJ, Berends CL, Akram N, Wezel J, Teeuwisse WM, Mik BG, Kan HE, Webb A, Beenakker JWM, Groeneveld GJ, (2017) Validation of a pharmacological model for mitochondrial dysfunction in healthy subjects using simvastatin: A randomized placebo-controlled proof-of-pharmacology study. European Journal of Pharmacology 815: 290-297. https//doi.org/<https://doi.org/10.1016/j.ejphar.2017.09.031>

18. Zhang H, Wang Y, Qu M, Li W, Wu D, Cata JP, Miao C, (2023) Neutrophil, neutrophil extracellular traps and endothelial cell dysfunction in sepsis. Clin Transl Med 13: e1170. https//doi.org/10.1002/ctm2.1170

19. Khalifa AR, Abdel-Rahman EA, Mahmoud AM, Ali MH, Noureldin M, Saber SH, Mohsen M, Ali SS, (2017) Sex-specific differences in mitochondria biogenesis, morphology, respiratory function, and ROS homeostasis in young mouse heart and brain. Physiol Rep 510.14814/phy2.13125

20. Sharawy N, Ribback S, Al-Banna N, Lehmann C, Kern H, Wendt M, Cerny V, Dombrowski F, Pavlovic D, (2013) Estradiol receptors agonists induced effects in rat intestinal microcirculation during sepsis. Microvascular Research 85: 118-127. https//doi.org/<https://doi.org/10.1016/j.mvr.2012.10.002>

21. Persson P (2019) Energie- und Wärmehaushalt, Thermoregulation. In: Brandes R, Lang F, Schmidt RF (eds) Physiologie des Menschen: mit Pathophysiologie. Springer Berlin Heidelberg, Berlin, Heidelberg, pp. 535-550

22. Yen TC, Chen YS, King KL, Yeh SH, Wei YH, (1989) Liver mitochondrial respiratory functions decline with age. Biochem Biophys Res Commun 165: 944-1003. https//doi.org/10.1016/0006-291x(89)92701-0

23. Müller-Höcker J, Aust D, Rohrbach H, Napiwotzky J, Reith A, Link TA, Seibel P, Hölzel D, Kadenbach B, (1997) Defects of the respiratory chain in the normal human liver and in cirrhosis during aging. Hepatology 26: 709-719. https//doi.org/10.1002/hep.510260324

24. Short KR, Bigelow ML, Kahl J, Singh R, Coenen-Schimke J, Raghavakaimal S, Nair KS, (2005) Decline in skeletal muscle mitochondrial function with aging in humans. Proc Natl Acad Sci U S A 102: 5618-5623. https//doi.org/10.1073/pnas.0501559102

25. Krishnan KJ, Greaves LC, Reeve AK, Turnbull D, (2007) The ageing mitochondrial genome. Nucleic Acids Res 35: 7399-7405. https//doi.org/10.1093/nar/gkm635

26. Larsson NG, (2010) Somatic mitochondrial DNA mutations in mammalian aging. Annu Rev Biochem 79: 683-706. https//doi.org/10.1146/annurev-biochem-060408-093701
